# A four-year medical school leader and leadership education and development program

**DOI:** 10.5116/ijme.5abe.12d2

**Published:** 2018-04-16

**Authors:** Erin S. Barry, Neil E. Grunberg, Hannah G. Kleber, John E. McManigle, Eric B. Schoomaker

**Affiliations:** 1 Military and Emergency Medicine, Uniformed Services University, Bethesda, USA

**Keywords:** Medical school leader, leadership, education and development program, USA

## Introduction

Leadership is a growing topic of interest in medicine and medical school education. The Association of American Medical Colleges now identifies leadership as “the most critical component for success”.^1^ Some medical schools in the United States are introducing leadership curricula, but there is a large gap that still needs to be filled.^2^ The Uniformed Services University of the Health Sciences educates and trains health professional leaders for the United States Army, Navy, Air Force, and Public Health Service. Leadership education has long been a part of this university’s mission. Recently, the university has expanded its Leader and Leadership Education and Development program that is provided to all Uniformed Services University medical students at the F. Edward Hébert School of Medicine.^3^ The purpose of this paper is to describe the comprehensive program and its conceptual framework that may be useful for other academic medical leader and leadership education and development programs around the globe.

### Leader and leadership education and development program

It is essential for leader and leadership education programs to define leadership; identify which students should become leaders; base the program on a conceptual framework; develop a curriculum consistent with the goals and conceptual framework; ensure that the program has a sound scholarly basis and include appropriate assessments.

Medical students are introduced to leadership and its relevance to medical education on their first day of medical school. Leadership is defined as influence on individuals and groups by enhancing behaviors (actions), cognitions (perceptions, thoughts, and beliefs), and motivations (why people act and think as they do) to achieve goals that benefit the individuals and groups.^4^^-^^6^ Leaders set the vision and inspire followers. The Uniformed Services University uses these definitions and delivers a four-year, comprehensive program as a requisite part of the undergraduate medical education of all medical students.

This program uses a variety of pedagogical techniques with emphasis on experiential learning. The program strives to develop adaptive leaders who are prepared to perform in volatile, uncertain, complex, and ambiguous environments.^7^^-^^9^ The program content and assessments are based on the FourCe-PITO Leadership Conceptual Framework. The four major elements of leadership (Four C elements or FourCe) are Character (who the person is), Competence (what the person does), Context (when and where actions occur), and Communication (how information is sent and received). These elements interact with each other and operate across four levels of psychological and social interactions – Personal (the individual), Interpersonal (between individuals), Team (small groups), and Organizational (large groups and systems).^4^^,^^10^

The curriculum is carefully woven throughout all four years of undergraduate medical education focusing on the formation of professional identity, acquisition of relevant knowledge and skills, and learning through experience. The topics of study pertain to leadership, such as crisis communication, effective communication, followership, the performance of individuals and teams under stress, personality and emotional intelligence, self-assessment and peer support, and team building.  Each session is based on at least one element of the FourCe-PITO framework. A variety of pedagogical styles and venues are used to deliver the curriculum, including interactive plenary sessions, flipped classrooms, small group exercises, group discussions, applied clinical and field settings. “Near peers” (more senior medical students) and core faculty facilitate small group discussions about leadership in healthcare settings drawing from recent and relevant experiences.

Leadership scholarship and research are foundational to this program. All concepts of leadership, management, and followership taught to the students are based on principles, theories, approaches, and techniques that have been developed by scholars and practitioners of leadership.^6^^,^^11^^,^^12^ The faculty conduct research and scholarship including evaluation of program effectiveness. Medical and graduate students have the option to participate in research projects. Current projects include gender and leadership; intergenerational leadership; development of leadership assessment tools; innovative teaching strategies.^13^^-^^16^

The program includes assessment of students’ knowledge and performance, faculty knowledge and performance, and program effectiveness. Assessments of leadership are based on quizzes; self-reflection; formative and summative feedback in applied settings; and faculty, peer, self-assessments using the FourCe-PITO framework.

Quizzes and self-reflection questions are given after each session, and students provide programmatic feedback. Results from the quizzes, self-reflection responses and feedback are used to improve sessions. The program has developed a self-assessment and peer support tool that is available as a smartphone app to help teach and promote self-awareness. Students are provided with their own formative ratings in each of the leadership elements as well as ratings based on input from several peers. This information enables students to see how they rate themselves compared to how their peers rate them on elements within the leadership framework. Additionally, all students participate in several medical field exercises over the four years where faculty evaluates them in applied settings.

All medical students in the program become physicians in the U.S. military or Public Health Service for at least eight years after graduation (and typically remain in these uniformed services for 10 - 25 years). Therefore, the program can assess performance as medical leaders for many years after completion of the medical school’s leadership program. This information is gathered and evaluated as part of the university’s Long-Term Career Outcome Study.^15^^,^^16^ Leadership elements in the database include student demographic information as well as performance during pre-clerkship, clerkship phases of education, and beyond. The merging of these data sets allows for evaluation of leadership performance in relationship to individual differences and medical professional performance.

## Conclusions

Leader and leadership education and development are essential aspects of medical education and are gaining attention in the United States. Medical schools should determine whether, who, how, when, and what should be taught about leadership. The Uniformed Services University provides a comprehensive, four-year leader and leadership education and development program for a medical student that may be valuable for other medical schools around the globe.

### Conflict of Interest

The opinions and assertions contained herein are the sole ones of the authors and are not to be construed as reflecting the views of the Uniformed Services University of the Health Sciences or the Department of Defense.
